# Ensemble modeling of auditory streaming reveals potential sources of bistability across the perceptual hierarchy

**DOI:** 10.1371/journal.pcbi.1007746

**Published:** 2020-04-10

**Authors:** David F. Little, Joel S. Snyder, Mounya Elhilali

**Affiliations:** 1 Department of Electrical and Computer Engineering, Johns Hopkins University, Baltimore, Maryland, United States of America; 2 Department of Psychology, University of Nevada, Las Vegas; Las Vegas, Nevada, United States of America; NTT communications Science Laboratories, JAPAN

## Abstract

Perceptual bistability—the spontaneous, irregular fluctuation of perception between two interpretations of a stimulus—occurs when observing a large variety of ambiguous stimulus configurations. This phenomenon has the potential to serve as a tool for, among other things, understanding how function varies across individuals due to the large individual differences that manifest during perceptual bistability. Yet it remains difficult to interpret the functional processes at work, without knowing where bistability arises during perception. In this study we explore the hypothesis that bistability originates from multiple sources distributed across the perceptual hierarchy. We develop a hierarchical model of auditory processing comprised of three distinct levels: a Peripheral, tonotopic analysis, a Central analysis computing features found more centrally in the auditory system, and an Object analysis, where sounds are segmented into different streams. We model bistable perception within this system by applying adaptation, inhibition and noise into one or all of the three levels of the hierarchy. We evaluate a large ensemble of variations of this hierarchical model, where each model has a different configuration of adaptation, inhibition and noise. This approach avoids the assumption that a single configuration must be invoked to explain the data. Each model is evaluated based on its ability to replicate two hallmarks of bistability during auditory streaming: the selectivity of bistability to specific stimulus configurations, and the characteristic log-normal pattern of perceptual switches. Consistent with a distributed origin, a broad range of model parameters across this hierarchy lead to a plausible form of perceptual bistability.

## Author summary

Our ability to experience the everyday world through our senses requires that we resolve numerous ambiguities present in the physical evidence available. This is accomplished, in part, through a series of hierarchical computations, in which stimulus interpretations grow increasingly abstract. Our ability to resolve ambiguity does not always succeed, such as during optical illusions. In this study, we examine a form of perceptual ambiguity called bistability—cases in which a single individual’s perception spontaneously switches back and forth between two interpretations of a single stimulus. A challenge in understanding bistability is that we don’t know where along the perceptual hierarchy it is generated. Here we test the idea that there are multiple origins by building a simulation of the auditory system. Consistent with a multi-source account of bistability, this simulation accurately predicts perception of a simple auditory stimulus when bistability originates from a number of different sources within the model.

## Introduction

Perceptual bistability—the spontaneous, irregular fluctuation of perception between two interpretations of a stimulus—can occur while observing ambiguous stimulus configurations [1–7]. A classic example of bistability is the Necker cube [1]. This image, comprised of an abstract line-drawing of a transparent 3D cube, can be interpreted as a cube seen from above or from below. If an individual stares at the cube, over time, perception will fluctuate between the above- and the below-view interpretations. In the auditory system, a prominent example of bistability comes from the classic auditory streaming paradigm [8, 9], in which a repeating A-B-A pattern of pure tones appears to be one (ABA) or two (A-A, and -B-) objects [4]. Here, we test the idea that perceptual bistability is generated from multiple sources distributed throughout the brain [3, 10–20], using a large ensemble of computational models of this auditory streaming paradigm.

The study of perceptual bistability, and the more general phenomena of multistability, has the potential to shed light on a number of fundamental principles of perception, as evidenced by four properties. First, its manifestation is characterized by substantial individual variation [21–32] providing a potential means for understanding how perceptual function differs across individuals. Second, it is a quite general phenomenon, as there are many forms of ambiguous stimuli that can generate perceptual bistability or multistability [1–4, 6]. These include not just abstract, laboratory stimuli, but also complex stimuli such as speech [6, 23, 33, 34] and faces [3]. Third, the proposed mechanisms of multistability are also candidate mechanisms for decision-making [18, 35–37] and perceptual inference [5, 7, 18]. Fourth, bistability provides a case in which perception varies while the observed stimulus remains constant, a valuable control when testing theories of attention, awareness and consciousness [38–41].

A number of properties of bistability can be accounted for using three simple ingredients: adaptation, inhibition and noise [18, 19, 42–50]. In isolation, inhibition can be used to define a set of attractors of neural activity; this in turn can lead to a winner-take-all behavior. Activity can shift from one of these attractors to another due either to noise or due to adaptation of the winning attractor. Appropriate forms of these dynamics can in turn be used to implement a number of fundamental computational elements necessary for decision-making [18, 35–37] and perceptual inference [5, 7, 18]. While these ingredients appear to reflect quite fundamental neural-computational principles, the actual impact on perception more broadly cannot be determined without knowing where bistability arises during perception.

An emerging idea is that, rather than originating from a single source, perceptual bistability is driven by many different sources of adaptation, inhibition and noise across the brain, in a distributed fashion [3, 10–20, 23, 33]. One reason this idea has been invoked is to account for an apparent contradiction in the literature: there is evidence which appears to favor an early locus [51–55], whereas other evidence suggests a much later locus [56–63] for perceptual bistability. A natural resolution to these differing outcomes is that there are multiple distributed sources of bistability [3, 11–17, 23, 33]. Another source of evidence for this distributed account comes from a careful examination of the distribution of reported percept lengths—the pattern of switches from one percept to another—which appear to be best explained by multiple bistable sources [19, 20].

If perceptual bistability really emerges from multiple sources across the perceptual hierarchy, then it should be possible to induce a plausible form of perceptual bistability within a model of this hierarchy. Yet, past efforts to model perceptual bistability do not systematically vary the locus and the magnitude of adaptation, inhibition and noise within a perceptual hierarchy. This makes it difficult to assess the relative merits of different sources of bistability within that perceptual hierarchy, as there are many confounding differences across computational models. Most mathematical models have focused on a single level of processing upon which adaptation, inhibition and noise generate an alternating response [18, 42–48, 50, 64–66]. The most relevant efforts to date, which include multiple hierarchical levels of processing [11, 13, 14, 17, 41], do not systematically vary the configuration of multiple loci of adaptation, inhibition and noise. Those efforts that do examine systematic variation of the parameter space [15, 18, 19, 41, 48, 50] consider only a single locus for bistability. An important reason for these limitations is that these past models have generally employed a relatively simplified description of perceptual input.

To begin to address these limitations, in the present report we evaluate a series of detailed hierarchical models of human behavior during a simple bistable auditory streaming task [4, 8, 9]. Our aim is an initial integration of auditory modeling of scene analysis with the modeling of perceptual bistability. As such, the present report focuses on the evaluation of the model for a well studied stimulus. The long-term goal is to integrate many bistable stimuli into this framework. The model includes three hierarchical stages: *Peripheral*, *Central* and *Object*. The Peripheral stage performs a time-frequency analysis; this is followed by the Central analysis which computes spectral features found more centrally in the auditory system. In the third, Object analysis, these features are bound into a probabilistic interpretation of the acoustic streams (or objects) present in the scene. Each model can be understood as some variant of an abstraction of the ventral auditory system [67, 68], in the sense that it generates an interpretation of what sources are present in an auditory scene, and does so in a manner consistent with principles of scene analysis gleaned from past empirical data [8, 9, 69–71].

Across the three stages of this hierarchy, we systematically vary the magnitude of adaptation, inhibition and noise within a model ensemble. Ensemble modeling may refer to a variety of somewhat distinct methodologies [72–76], all of which leverage the advantages of evaluating model dynamics across a large parameter space. In our cases many of the evaluated model parameters may fail to reflect human behavior, but a subset provide a good fit to the empirical data. This approach avoids the assumption that a single parameter configuration must be invoked to explain all of the experimental data [72, 73]. Rather, there may be many models within the ensemble capable of accounting for the available data, reflecting variation that may occur both within and across individuals.

Our focus here is on evaluating each model configuration’s ability to capture several key behavioral hallmarks of perceptual bistability in a population of human listeners, relative to the ability of individual human listeners to capture these same hallmarks. We have opted for this behavioral metric for model evaluation, rather than examining various analogues of a neural signature. We made this choice because of the implications of a distributed origin for perceptual bistability: if bistability is caused by a number of distinct, distributed sources, there may be no localized neural signature within these progenitors. Instead, the perceptual manifestation could arise from an interaction across multiple loci. In this case the most direct neural correlates to bistability would only arise during a “read-out” of the original progenitors of bistability.

Our approach of varying the magnitude of adaptation, inhibition and noise across the three stages allows us to focus on the key question of interest here: where these three components can plausibly lead to perceptual bistability across the perceptual hierarchy. It specifically allows us to identify where the presence (magnitude far from zero) or absence (zero or near zero magnitude) of these components within our model is best supported by human data. As such the focus here is not on a quantitative fit of the model’s temporal dynamics—these can vary substantially across listeners [21, 25, 77]—but rather, on the overall pattern of responses, and the robustness of this pattern to changes in the other parameters of the model (e.g. time constants).

When we systematically vary the level of adaptation, inhibition and noise across the auditory hierarchy, we find that a plausible form of bistability can be generated in the output of the model by using these three ingredients at any one of the three different stages of analysis or across all stages simultaneously. This finding demonstrates that each stage can contribute to bistable perception, and that they can all be active simultaneously. The results are therefore consistent with a multi-source hypothesis for perceptual bistability. Furthermore, the range of levels of adaptation and inhibition that generate a plausible form of bistability is much larger at the highest stage of this hierarchy (Object), while a much more precise tuning of adaptation and inhibition is required at the earliest stage of analysis (Peripheral)—and the Central-analysis parameter tuning is somewhere in-between these extremes. These differences in parameter sensitivity across the model hierarchy suggest that, while distributed, object-level sources of bistability may predominate. These differences in sensitivity could also, perhaps, be an indication that there is more individual variation in sources of bistability at later stages of analysis, given the considerable individual variability of bistable perception [21–32].

## Results

### Model description

**Fig 1:**
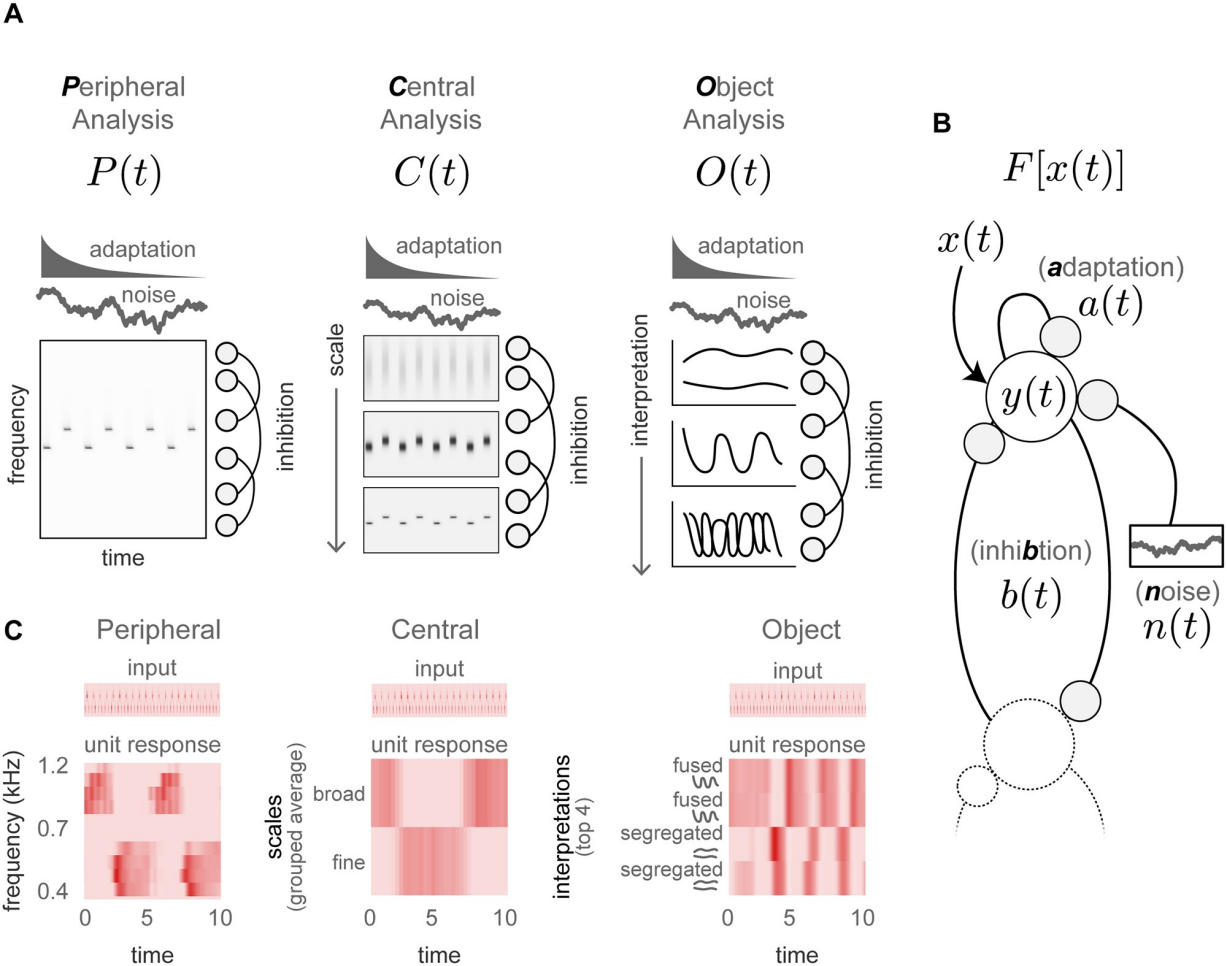
Model design. Each model in the ensemble includes three hierarchical analysis stages: Peripheral, *P*(*t*), Central, *C*(*t*), and Object, *O*(*t*), over which we apply the same form of adaptation, inhibition and a small amount of noise, *F*[*x*(*t*)] **A**. The three analysis stages. The Peripheral analysis, *P*(*t*), computes a log-frequency spectrum using cochlear-like filter shapes. The Central analysis, *C*(*t*), computes multiple spectral scales of *P*(*t*), to capture dependencies across different frequencies. The Object analysis, *O*(*t*), computes multiple interpretations of the auditory scene into separate masks of *C*(*t*), selecting the interpretation most consistent with the data at each time frame. **B**. The application of adaptation, inhibition and noise. Adaptation, *a*(*t*), reduces the input, *x*(*t*), by a low pass version of the output history; inhibition, *b*(*t*), reduces the input by a low pass version of the output history of distant neighbors; noise, *n*(*t*), adds a small amount of variation to the output weights. The result is a set of output weights, *y*(*t*), applied within each model along the frequencies (for Peripheral), scales (for Central) or scene interpretations (for Object). **C**. Illustration of model unit responses to a given auditory streaming input when adaptation, inhibition and noise are applied to the given stage, and only that stage. The Central units have been averaged across the lower and upper half of the scales. The Object units have been selected from the top 4 possible interpretations, along with an approximate description of the given interpretation.

Fig 1A shows our computational framework: it includes three levels of processing: Peripheral (Fig 1A; left panel), Central (Fig 1A; middle panel) and Object (Fig 1A; right panel). Within each of these levels, we apply a given amount of adaptation, inhibition and noise, shown in Fig 1B. Full details of the model design can be found in Materials and methods. Overall the results show that a bistable response—the alternations of the model output—emerge with the introduction of adaptation, inhibition and noise in any one of these three stages independently, or when all three stages include these three terms. Furthermore, it generates a bistable response across the broadest range of variations of adaptation and inhibition in the Object-level stage of analysis.

During the Peripheral analysis stage (Fig 1A; left panel) the model computes a time-frequency representation of the sound. This analysis resembles a typical short-time Fourier transform, but includes several features which are more biologically plausible for the auditory periphery: this includes log-frequency cochlear-shaped filters, half-wave rectification and firing rate limitations.

During the Central analysis stage (Fig 1A; middle panel) the model computes a series of time-frequency analyses that vary in their spectral scale. Each scale is defined by a series of band-pass filters, all with the same width, applied along the dimension of log-frequency of the auditory spectrogram from the Peripheral analysis. Spectral scale captures the spectral dependency of measured receptive fields found in the inferior colliculus (IC) [78–81] and primary auditory cortex (A1) [69, 82, 83]. For our purposes in this report, we focused on this single set of frequency-based features to limit complexity during the systematic exploration of a large ensemble, though it could conceivably be extended to any number of additional relevant perceptual features.

During the Object analysis stage (Fig 1A; right panel) the model computes multiple interpretations of the acoustic scene. Each interpretation consists of one or more groupings of Central features into streams (or objects). The different scene interpretations emerge on the basis of the hyper-parameters of this stage, which vary in value from levels that imply rapid changes over time, to gradual changes over time. These varying scene interpretations are weighted on the basis of their posterior probability. Consistent with the evidence concerning the formation of auditory objects in the brain, these features are grouped through a process of temporal coherence [8, 9, 70, 84, 85] and strung together over time on the basis of object continuity [9, 71, 86, 87]. Temporal coherence means that the formation of initial groupings depends on the short-term correlations in the scene across different time scales: as such, acoustic events that occur simultaneously or nearly simultaneously will be grouped together. Object continuity means that events grouped during temporal coherence that appear similar across time are grouped together as one object. These two stages were selected with the goal of showing a range of plausible grouping behaviors across the stimulus of interest here (the A-B-A repeating tones), while adhering relatively closely to existing principles of scene analysis. The temporal-coherence stage has proven effective on its own in existing work [70] within a similar framework. We required the additional, second object-continuity stage to operate across the longer time scales necessary to examine perceptual bistability.

For the present, auditory streaming stimulus, the two components of the Object-level analysis play complementary roles: first, temporal coherence allows us to identify two distinct clusters of responses in the output of the central analysis, corresponding to the two tones (A and B) from the multitude of frequency-scale outputs of the Central stage. Second, the object continuity stage allows these two tones to either be treated as two separate streams or grouped into a single stream, depending on the prior. Over the course of the stimulus the final posterior probability of these two possible scene interpretations changes, due both to evidence accumulation and to fluctuations in the strength of different interpretations caused by adaptation, inhibition and noise.

We vary the behavior of these three stages across a large ensemble of models by systematically varying the magnitude of three terms—adaptation, inhibition and noise (Fig 1B)—across the three analysis stages. These terms are applied to a set of weights which determine the relative strength of different frequencies (Peripheral), scales (Central) or scene interpretations (Object). Adaptation reduces each weight by a delayed, low-pass version of itself (self-referential link in Fig 1B). Inhibition reduces each weight by a delayed, low-pass version of neighboring weights (lines to and from second unit in Fig 1B). Noise modulates each weight randomly (line to noisy response in Fig 1B). These basic components have been applied in various forms throughout the years to model perceptual bistability [18, 19, 41–43, 47–50].

Fig 1C provides a simplified illustration of the response of each analysis stage to the input. It aimes to provide some intuition of the effects of adaptation, inhibition and noise within the model. Each output shows how the given stage responds when adaptation, inhibition and noise are applied to that stage, and that stage only. For ease of interpretation we have opted to simplify the unit responses for the Central stage and Object stage outputs: the Central stage is averaged over all frequencies, and over multiple scales—grouped into the lower and upper halves—and the Object stage includes a set of the most probable interpretations and a schematic representing the approximate scene configuration. From this panel it can be seen that adaptation, inhibition and noise can induce distinct periods of alternating activity at each stage of the model. Fig 1C also provides a demonstration of an important principle of distributed bistability—that the earlier alternations of unit strength do not directly correspond to bistable perception, but instead are interpreted by the Object stage and ultimately generate a bistable response.

### Model evaluation

**Fig 2:**
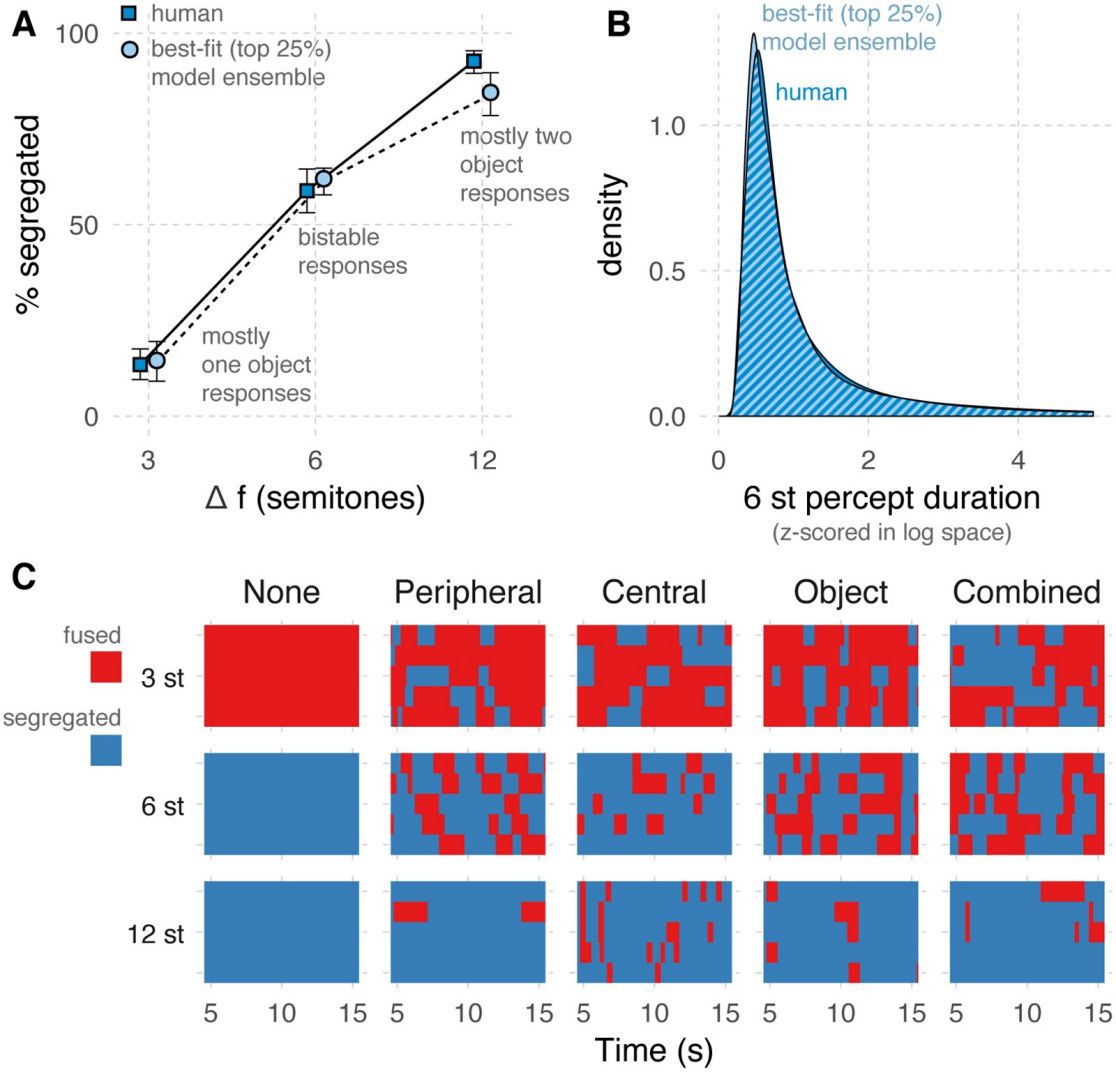
Best-fitting models. The performance of humans and models that best fit human behavior. Models included here fell in the top 25^*th*^ percentile according to an aggregate measure of model fitness (the model:human deviation ratio). **A**. The percent of segregated (2 or more object) responses. Human data were taken from two existing studies [16, 88]. Error bars denote the 95% confidence intervals estimated by bootstrap. Human data include a total of N = 72 participants. **B**. The distribution of response lengths for the 6 semitone stimulus across all listeners, and the best fitting models. The lengths of each individual model and human listener were first Z-scored in log space. Human data include a total of N = 35 participants, with an average of 211 samples per listener (minimum = 20, maximum = 1232). Percept lengths were combined across the two percept types: fused and segregated. **C**. Individual data for 5 example models (columns) across the three stimulus conditions (rows). The “None” model includes no adaptation, inhibition or noise. The “Peripheral”, “Central”, and “Object”, include adaptation, inhibition and noise in their respective levels, and the “Combined” model includes these terms in all three stages. Each panel represents periods of fused (red) and segregated (blue) responses over five simulation runs (y-axis) of a 10-second time period during the steady state response of the model output (x-axis).

To evaluate each model in our ensemble we compare its ability to predict human behavior on two data sets, shown in dark blue in the panels of Fig 2; together these data sets capture two key hallmarks of perceptual bistability.

The first hallmark of bistability is that it is selective to stimulus ambiguity: for the A-B-A stimulus employed here this means that bistability is most pronounced at an intermediate (6 semitone) frequency separation. Fig 2A shows human data which captures this hallmark [16, 88]. It depicts behavior in terms of the percent of segregated responses (y-axis), across the three stimulus conditions tested (x-axis). A segregated response is one that indicates the listener heard two or more sounds, and a fused response indicates only one sound was heard. The pure tones in the ABA pattern presented to listeners were separated by either 3, 6 or 12 semitones (st). Only the 6 st stimulus elicited a clearly bistable response: listeners reported the 3 st stimulus was mostly heard as a single sound source, and the 12 st stimulus as mostly two streams of sound.

The second hallmark of bistability is the characteristic distribution of response lengths. The response length is the amount of time between when a listener reports hearing one stream and when they report hearing two streams of sound, and vice versa. Fig 2B shows human data which captures this hallmark (c.f. Human data). These data depict behavior in terms of the distribution (y-axis) of Z-scored response lengths (x-axis), but only for the most ambiguous stimulus (6st). The figure shows that there were periods of relatively stable perception (the distribution has a long tail), with some characteristic length (the distribution peaks above zero). Z-scores were applied to each individual configuration of the model and each human listener in log space. This normalization is applied because (1) the rate of switching varies across individual listeners [21, 25, 77], and (2) our focus in this study is on evaluating model fit to the overall pattern of responses, rather than evaluating a quantitative match to the temporal dynamics of individual listeners. We also find that there is a similar match between model and data when the lengths are mean-normalized (following the normalization used in [4]).

We chose to use different human data sets across these panels because neither data set could capture the characteristics of the other: in the first two studies [16, 88] the trials were too short to avoid cutting off the tail of the distribution shown in Fig 2B, and so the pattern of response lengths could not be determined; the data presented in Fig 2B focused on bistable responses, using only the 6 st stimulus, and employed longer trials; these data did not include the 3 or 12 st stimulus.

To evaluate each model’s ability to capture the first hallmark of bistability we are interested in—the selectivity of bistability—we compute a quantity referred to as the *response deviation*: the deviation of model predicted responses from that of the mean human listener. Specifically, for each stimulus (3, 6 and 12 st), we found the proportion of “segregated” responses (ala Fig 2) for each individual simulation run and for the average human listener, and then computed the root-mean-squared difference between model and human responses across all three stimuli.

To evaluate each model’s ability to capture the pattern of response lengths, we compute the *response-length deviation*: across all simulations of a given model we compute the Kolmogorov-Smirnov statistic [89] of the model’s distribution of Z-scored response lengths vs. that same distribution for the human listeners. We selected the Kolmogorov-Smirnov statistic—the maximum difference between the empirical cumulative distribution function of two samples—for its sensitivity to subtle differences between two distributions.

These two measures of deviation are combined to provide an overall fitness score for each model, referred to as the *model:human deviation ratio*. To accomplish this, the two deviation measures are first placed on a comparable scale by dividing model deviation by the deviation of individual human listeners. The two deviation ratios are then averaged. We compute deviation for each individual human listener from the overall sample using the same procedure described above for computing model deviation. Specifically, each individual’s response deviation is the root-mean-squared difference from individual to mean proportion of “segregated” responses. An individual’s response-length deviation is the Kolmogorov-Smirnov statistic of their Z-scored response lengths compared to the Z-scored response lengths of all listeners.

To provide some intuition about this measure: the larger the model:human deviation ratio, the greater the model deviation is relative to the deviation of the average human listener from the mean. A deviation of 1 would indicate the model has the same amount of deviation from the mean as the average individual human listener.

To determine whether our ensemble includes models that are consistent with human behavior, we examine the behavior of the top 25% of models, according to this model:human deviation ratio (light blue circles in Fig 2A; light blue region in Fig 2B). These model data support the merits of our aggregate measure, confirming that when models score well (low) by this measure, they collectively show behavior quite similar to the mean human data.

As a further check on model behavior, we examine several representative models in more detail (Fig 2C): one including no adaptation, inhibition or noise, one including these three terms in just the Peripheral-level, just the Central-level and just the Object-level analysis, and a final model with the three terms in all three levels simultaneously. When the Peripheral level includes these terms, their magnitudes are set as *c*_*a*_ = 15 (adaptation) and *c*_*b*_ = 130 (inhibition). For Central and Object, their magnitudes are set as *c*_*a*_ = 5 (adaptation) and *c*_*b*_ = 5 (inhibition). These are selected from the top 25% of the ensemble for the within-stage model variations (discussed in Model behavior across the ensemble below), and such that their combination yields a reasonable model for the Combined example (informed by the across-stage model variations, also discussed below).

In Fig 2C the individual behavior for 5 simulation runs are shown. This behavioral output was generated by a thresholding of the time-frequency-mask output of the Object level stage (see Interpretation of model output). Each panel in this figure represents whether a model reports a fused (red) or segregated (blue) response at the given time (x-axis) and simulation run (y-axis), across the three different stimuli tested (rows). This figure displays the reported percepts of a model simulation, starting at 5 seconds to avoid the “buildup” phase of the responses (c.f. S1 Fig). The response to a given stimulus is constant for the “None” model. The remaining four models demonstrate the most balanced alternations for the 6 st stimulus, and show less ambiguous, but still bistable responses for the 3 st and 12 st stimuli, consistent with the indications that these stimuli can also include some bistable fluctuations [90]. This figure demonstrates that the inclusion of adaptation, inhibition and noise, at each individual analysis level, or at all levels at once can generate reasonable bistable behavior.

In a supplementary figure (S1 Fig), we also examine the early responses of the same five models from Fig 2C: specifically from 0 to 10 seconds. For each model with some amount of adaptation, inhibition and noise, we find a rough qualitative match to a phenomenon sometimes referred to as “buildup” (e.g. [4, 91, 92]): a consistent transition from “fused” responses at the start of a run to a more average balance between “fused” and “segregated” response. The results are fairly coarse, showing just a qualitative match to the human outcomes, and some deterministic behavior in early alternations of the Object stage analysis. These outcomes are representative of the buildup curves of other models we examined, though not all show this buildup property. Since we were focused on the magnitudes rather than the time constants of adaptation, inhibition and noise, we did not seek out a more precise temporal fit to the human data. Though we find only a rough qualitative match, it remains reassuring that the model, not specifically designed to handle buildup, is able to show an outcome reminiscent of buildup.

### Model behavior across the ensemble

We now evaluate the behavior of the entire model ensemble, using the aggregate model:human deviation ratio and the two separate deviation measures—response and response-length deviation (see Model evaluation). We focus on the systematic variation of adaptation and inhibition both within and across the levels of the hierarchy. Varying the amount of noise across levels did not appear to change the key conclusions of our results (see Sensitivity analysis).

**Fig 3:**
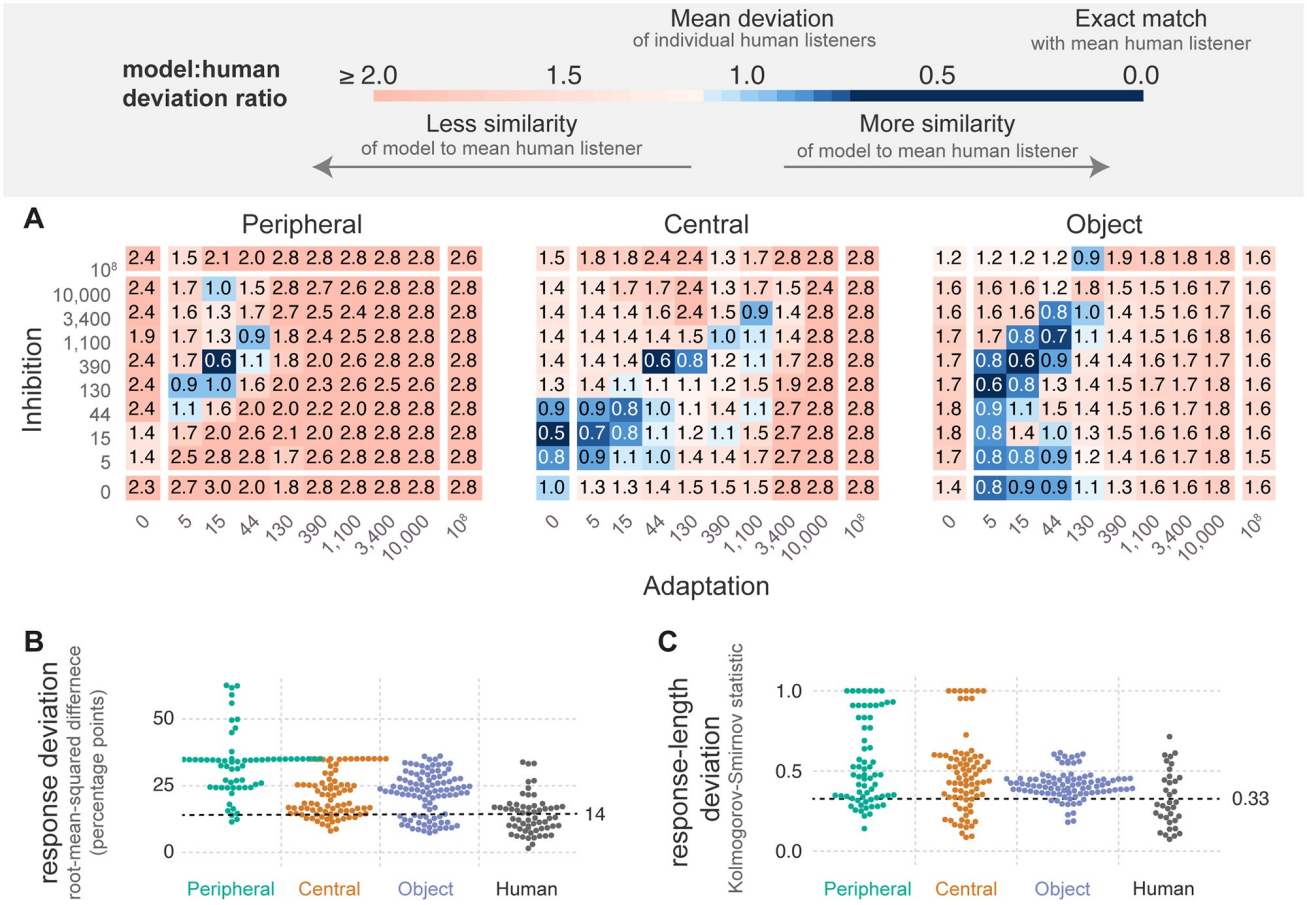
Within-stage variations of adaptation and inhibition. Each model shown includes terms for adaptation, inhibition and noise in one of the three analysis stages, leaving the other two stages with no such terms. This configuration lead to a total of 3 × 10^2^ = 300 model variants corresponding to the 3 perceptual stages, with 10 different levels of adaptation and inhibition. **A** The model:human deviation ratio for all levels of inhibition and adaptation at each of the three analysis stages. Each square represents average deviation for all simulations of a model with the given level of adaptation and inhibition. Values above one (pale blue to red) indicate models with more deviation than the average human listener; values below one (blue colors) indicate models with the same or less deviation than the average human listener. These measures are averaged across the ratio of both response deviation (panel B) and the response-length deviation (panel C). **B** The response deviation for all models and the human listeners from [16, 88] (N = 72). Each point for the three model variations represents the average response deviation for all simulations of a single model from the ensemble. Offsets from the central x-axis of each condition are used as a visual aid, to ensure that all data points are at least partially visible. The dotted line marks the mean of the human data: the deviation ratio is greater than one above this line and less than one below this line. **C** The response-length deviation for all models and human listeners. Human data are the same as those shown in Fig 2B (N = 35).

Fig 3 shows the within-stage model variations of adaptation and inhibition: in these variations, adaptation, inhibition and noise are applied to only one of the levels of the hierarchy at a time, setting their parameters to zero for the other two stages.

Fig 3A shows behavior for the within-stage model variations in terms of the model:human deviation ratio. This measure is shown for each level of adaptation (x-axis) and inhibition (y-axis) tested within each of the three levels of the hierarchy (columns). The figure supports two key findings: that some variations in the parameters of each level lead to human-like model behavior (there is blue in all three panels); and more such variations of the higher-level model parameters than lower-level parameters lead to human like behavior (there is more blue moving from left to right).

The overall outcomes reflected by the model:human deviation ratio are also supported when we examine the two deviation measures separately: response deviation—in Fig 3B—and response-length deviation—in Fig 3C. Note that points are displaced along the x-axis within this figure to ensure that each datum is visible; thus, the overall shape of this plot provides some indication of the distribution of the data. Both figures show that some individual models (data points) fall at or below the mean human deviation (dotted lines). Further, as we move up along the hierarchy, the mass of points (the mean) is lower, meaning that there are increasingly more models more consistent with the human data.

**Fig 4:**
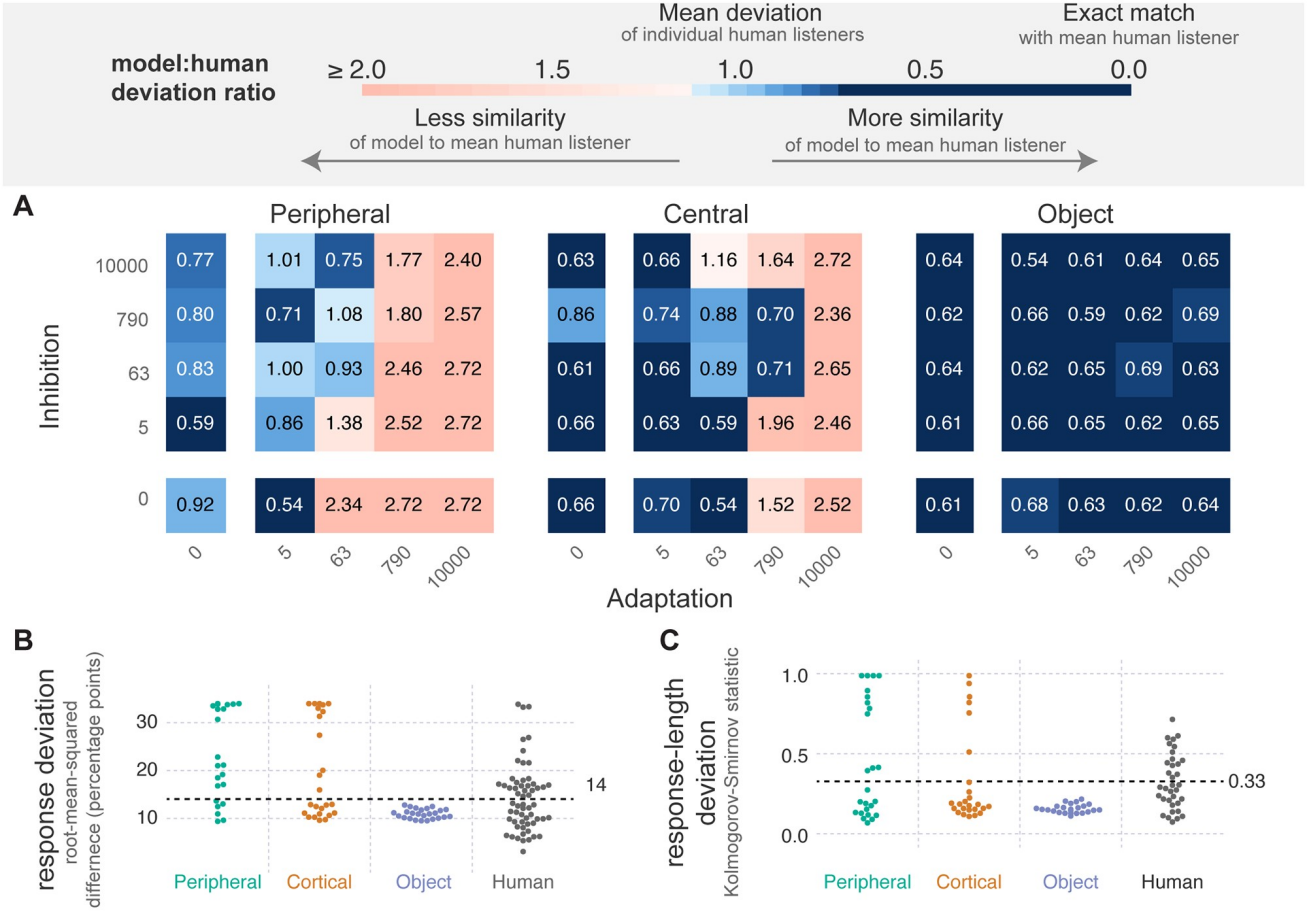
Across-stage variations of adaptation and inhibition. Each model in the ensemble includes some amount of adaptation, inhibition and noise at all three analysis stages. This configuration lead to a total of (5^2^)^3^ = 390, 625 model variants corresponding to the 5 different levels of adaptation and inhibition across the three analysis stages. **A** The minimum model:human deviation ratio for each value of adaptation and inhibition at each level. Each square represents the average for all simulations of the best performing (minimum deviation) model with the given level of adaptation and inhibition. Values above one (pale blue and red colors) indicate models with more deviation than the average human listener, values below one (blue colors) indicate models with the same or less deviation than the average human listener. **B** Response deviation for the data shown in A: that is, the minimum percent-streaming deviation for each pairing of adaptation and inhibition, tested at each level of the model hierarchy. The dotted line indicates the average deviation of the human listeners. **C** Response-length deviation for the data shown in A. Figure follows the same format as described for panel B.

Fig 4 summarizes the results of the across-stage variations of adaptation and inhibition: in these models, adaptation and inhibition are varied simultaneously across all three levels. Despite the presence of these terms across all three layers in many possible configurations, the results remain quite similar to the within-stage variations of Fig 3: there are non-zero levels of adaptation and inhibition at all three stages that lead to human-like behavior (blue in all three panels), and there are more such accurate model variations as we move up the stages of analysis (blue increases from left to right). Note that each datum in these figures (square in A or point in B and C) represents the best (minimum) mean deviation ratio across all models with the given magnitude of adaptation and inhibition at a specific analysis stage. Therefore, for a given stage, each point is representative of the minimum deviation ratio of 625 models (5^2^ × 5^2^), one model for each level of adaptation and inhibition at the other two analysis stages.

### Sensitivity analysis

**Fig 5:**
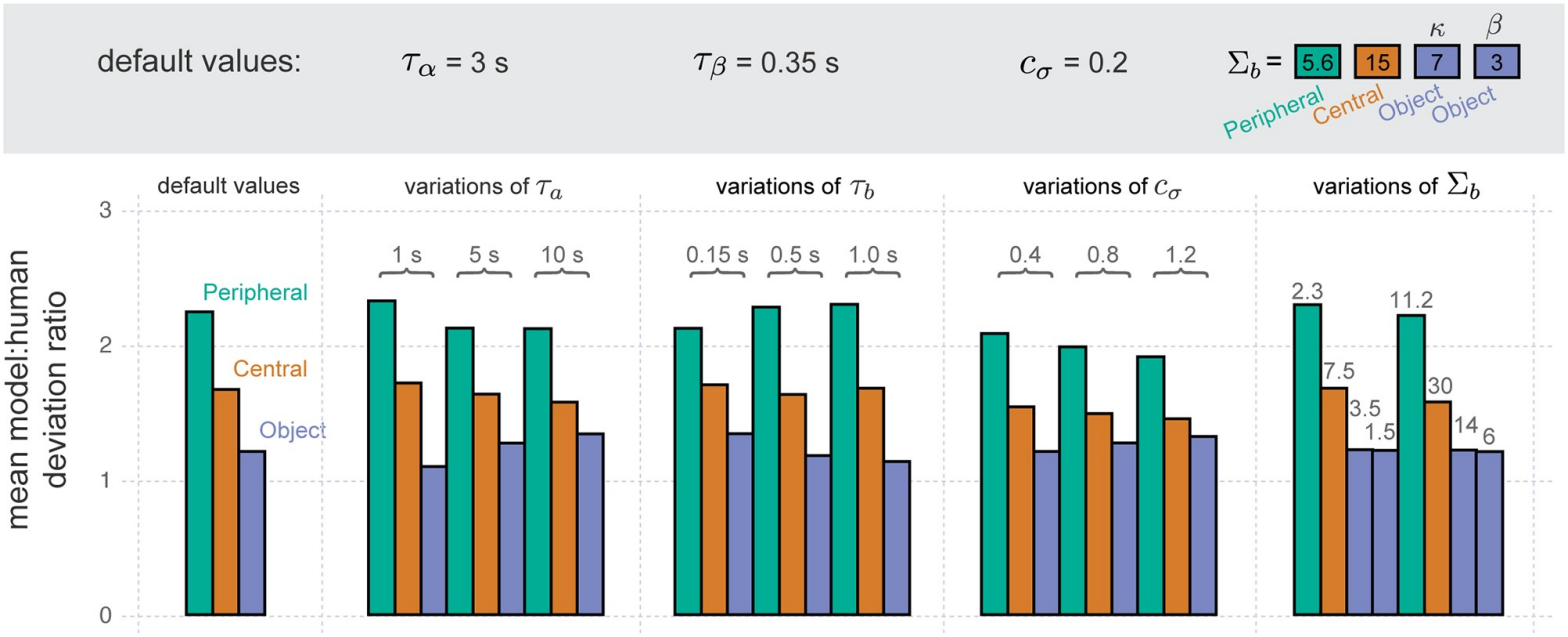
Sensitivity of model. A summary of the overall model behavior across different parameter variations. The default-parameter model essentially summarizes the results from Fig 3A, though the models tested here use a coarser grid of variations across adaptation and inhibition, consistent with the grid used for the remaining parameter variations shown in the present figure. For each parameter varied (columns), we consider multiple possible alternatives (numbers above each group of bars), changing it from a default value (shown above each group, within the gray region). Note that the values of inhibition breadth (Σ_*b*_) differ across the levels because their effect on behavior differs across the levels.

To determine how sensitive our results are to specific parameter values, we examine the effects of varying these parameters on the model:human deviation ratio (Fig 5). We vary the time constants for adaptation (*τ*_*a*_) and inhibition (*τ*_*b*_), the magnitude of noise (*c*_*σ*_), and the breadth of inhibition (Σ_*b*_). For each parameter considered we vary that parameter, while leaving all other parameters at their default value. For each parameter setting we examine the within-stage model variations, but using a coarser grid than shown for the within-stage model variations from Fig 3: instead we use the 25 parameter combinations shown for Fig 4. An inspection of the individual data revealed no large differences between the results, regardless of the parameter varied. (That is, we re-plotted Fig 3 for all 13 model variants)

Fig 5 provides a summary of all model variants. It shows the mean model:human deviation ratio (y-axis) for all the within-stage model variations when using the default model parameters (far left column) and when using a number of different parameter variants (x-axis). For ease of reference, the default model parameters are also shown (upper gray row). Note that the final parameter (Σ_*b*_) had distinct values within each level of the hierarchy because its meaning depends on the number of competing units, which varied across level.

This figure shows that one of the key results of our analysis is not altered by variations in the model parameters. Regardless of parameter variant, more variations of adaptation and inhibition are consistent with the human data as we move up the hierarchy. As such, the mean model:human deviation ratio across the variations of adaptation and inhibition decreases as we move up the hierarchy.

Furthermore, all of these parameter variants have some model variations consistent with the human data. The average minimum model:human deviation ratio across these variants is 0.88 (SD = 0.18).

## Discussion

In the model ensemble reported here, each model includes three levels of processing—Peripheral (Fig 1B; left panel), Central (Fig 1B; middle panel) and Object (Fig 1B; right panel). We systematically vary the amount of adaptation, *a*(*t*), inhibition, *b*(*t*) (Fig 1B) and noise, *n*(*t*), within each level separately (Fig 3), and across all levels simultaneously (Fig 4). We then compare model responses to past reports of human responses to a pure-tone ABA pattern; human listeners were asked to respond continuously, indicating whether they heard a “fused” (1 object) or “segregated” percept (2 or more objects).

There are two key findings: (1) there are models consistent with human behavior regardless of the locus of bistability we consider here, either within each analysis stage (Fig 3 shows blue in all three panels) or across all three simultaneously (Fig 4 shows blue in all three panels); (2) the number of variations of adaptation and inhibition generating plausible bistability increases as the locus of bistability within the perceptual hierarchy increases. This increase in viable model variation up the hierarchy is true both for the within-stage variations (Fig 3; more blue to the right) and for the across-stage variations (Fig 4; more blue to the right). Note that the standard for “plausible” in this case is that a model predominantly generate perceptual bistability for the intermediate (6 st) stimulus of the three stimuli presented to the model (3, 6 or 12 st; Fig 2A) and that the pattern of perceptual switching follows the systematic, log-normal-like distribution characteristic of perceptual bistability (Fig 2B). These results appear relatively robust, as they are not substantially altered by variations of several model parameters (Fig 5).

From these two model results there are three possible implications for auditory function. First, the ability of all three stages to contribute to bistability both in isolation and simultaneously suggests that perceptual bistability could be pervasive, arising from many levels of the auditory hierarchy in a distributed fashion. Second, it is possible the observed object-level dominance implies that, while distributed, object-level analyses play a more predominant role in generating bistable perception. Third, when the dominance of the object-level model variations is considered in light of substantial individual differences in perceptual bistability [24–32], this model predicts that such individual variations would occur predominantly at later, more object-level stages of analysis, and less at early, peripheral stages.

### Distributed sources of bistability

The notion that perceptual bistability may emerge from many different stages of analysis has received converging support from a number of different sources [3, 10, 12–14, 16–20, 48, 50–63]. However, existing efforts to model the locus of bistability across a hierarchy [11, 13, 14, 17, 41] differ from the present effort in that they (1) do not consider multiple possible loci of bistability within this hierarchy and (2) do not introduce adaptation and inhibition within a system that actually computes a scene interpretation from raw sensory input. These two differences strengthen the support for the pervasiveness of perceptual bistability because they show that (1) bistability can emerge from a large variety of different model configurations and that (2) this emergence can scale from more idealized models to a more complete system capable of generating perceptual inferences relevant to behavior. Outside the region of an ambiguous stimulus presented to our model (6 st), the model computes a useful mask that either fuses a scene into one stream (for 3 st) or extracts it into two streams (12 st).

Past computational studies of bistability have focused on a single locus at either relatively early stages [65, 66] or late stages of processing [64, 93]. None of them systematically vary the locus. In Rankin et al [65] (and also [66]), a relatively low-level source of auditory bistability is examined: three broadly tuned frequency channels compete and these can be used to explain bistability during the ABA tone pattern. This approach is similar in spirit to introducing bistability during the Central or Peripheral analysis stage of our model: in both examples there are a set of competing, broadly tuned tonotopic features, and there is no inference process which groups features into objects. In Mill et al [64] and in Barniv and Nelson [93] a higher-level source is evaluated, in which probabilistic interpretations of the acoustic scene compete with one another. The implicit assumption is that bistability occurs over some representation of auditory objects. In this way it is similar to introducing bistability during the Object stage of our model. Note that any one of these accounts of competitive dynamics might apply at an earlier or later stage of stimulus representation. Yet none systematically explore this possibility.

Existing bistable models of auditory streaming also represent input in a manner that is far more constrained than the present model: a small number of units represent the stimulus. This approach limits the ability to compare the merits of different bistable loci within a single system, because the transformations across stages of analysis are precisely when the details of how input is transformed from a raw acoustic input start to be important. Existing auditory models of this streaming paradigm that do include a more detailed perceptual hierarchy do not appear to demonstrate any form of perceptual bistability [70, 85, 94].

The present model can be understood as a hybrid of past approaches. It incorporates a form of adaptation, inhibition and noise, of a simpler form but similar in spirit to past competitive models [65, 66]; it incorporates a form of evidence accumulation during the Object-level analysis, akin to more probabilistic models [64, 93]; and furthermore, the competitive dynamics are included across a hierarchy, akin to past hierarchical perceptual models [70, 85, 94].

This modeling approach also provides an illustration of an important concept of distributed bistability: that the most obvious neural correlates of bistability may only represent a read-out of some earlier, more opaque progenitor of bistability. For example, the fluctuations in emphasis between units near A- and B-tone frequencies, shown in Fig 1C, do not directly match a specific scene interpretation, yet their presence is required to induce behavioral bistability (Fig 2C).

### A systematic increase of variation up the perceptual hierarchy?

The existing evidence for individual differences in perceptual bistability [5, 7, 18, 21–23, 25, 30, 32, 43, 47–50, 77, 95, 96] suggest that the breadth of functional variations represented by our model ensemble may also be present in the human population. That is, there may be more variation in the sources of bistability at higher levels of the perceptual hierarchy. Most likely, this observed pattern was due to the change in how the signal is represented at these stages. This intriguing prediction suggests that one promising line of future work would be to compare this model or its extension to human data at the individual level.

### Relation to physiology

Our analysis indicates that plausible forms of bistability can be generated by the dynamics of a number of distinct functional representations (peripheral, central and object), and that it is possible for all of these generators of perceptual bistability to exist simultaneously. To the extent that these representations are employed within a given physiological substrate, they could support perceptual bistability. The key point is that these are *functional* characterizations of the auditory system, rather than precise physiological analogues. Our conclusions rely on a systematic evaluation of the amount of adaptation, inhibition and noise present across many models. Other parameters of the simulation were not precisely tuned, and our key conclusions appear relatively robust to variations in these other model parameters. The model thus provides a qualitative assessment of how the magnitude of adaptation and inhibition across a perceptual hierarchy may induce bistable behavior. Therefore, appropriate comparisons between this model and physiology should be at the level of functional process, rather than any precise match between model parameters and physiology.

One natural mapping between the present model and physiology would be to relate the Peripheral stage to brainstem, Central to midbrain or primary cortex, and Object to primary and/or secondary areas of auditory cortex. Concordant with the idea that each area contributes to bistability, several long-term averages of neural activity are consistent with the buildup of auditory streaming within early brainstem (cochlear nucleus) [92], and cortex [91]. These sites could be showing an appropriate correlate for auditory streaming because each contributes, in a distributed fashion, to the final bistable report of perception.

If there are multiple generators of these bistable dynamics arising from these different loci, this could occur in a number of ways. Emergent properties of a population of neurons [97], the dynamics of sub-threshold membrane potentials [98], exploration-exploitation trade-offs in perceptual decision making [23, 59], short-term plasticity, predictive coding [7, 99], and top-down modulation [7, 41] may all have a role to play in placing the system in a state where these dynamics could emerge within each site, or across sites.

The dynamics of bistability would likely *not* arise solely from the intrinsic rates of adaptation and inhibition of isolated neurons, as these can be much shorter in the brainstem and midbrain [100, 101] compared to even the shortest time constants employed in the present case. However, the rates of stimulus-specific adaptation in the midbrain fall along a more realistic time scale [97, 102–105]. None-the-less, we would be wary of any direct analogies to single-unit recordings and the present model. How the population- and system-wide processing stages simulated here map to individual neurons remains unclear. One must be especially cautious given the possibility that, within a distributed system, the progeintors of bistable behavior may not show an obvious relationship to perceptual alternations; these alternations may only be apparent in the read-out these progenitors. A precise mapping from individual neurons to this model would require further careful study.

### Future work

Our focus in this report is on the magnitude of adaptation, inhibition and noise during auditory streaming at three stages of perceptual analysis. While this has proven fruitful, it necessarily excludes many other fascinating aspects of perceptual bistability.

First, there are a number of other plausible sources of bistability: alternations in top-down interactions [5, 7, 106], alternations in pathways outside the auditory system [23, 107], and alternations at multiple time scales [108]. Given the ability of the three forms of competition examined in the present model to co-exist within a single system, we speculate that these additional forms of competition may all co-exist to varying degrees.

Second, there are many details of the temporal dynamics of bistable models that we have omitted [18, 19, 48, 50, 65, 109–111]. The innovations considered from this past work, such as recurrent excitation [110, 111], noise-dominant dynamics [50, 109], multiple oscillators within the same level of analysis [19] or low-level background activity [18, 48], may be necessary to fully capture the dynamics of human data [2]. Future efforts along these lines will likely require additional measures of model performance that can assess the quantitative match to the time course of model and human behavior, such as a precise match to the “buildup” phenomena, rather than the very coarse qualitative match found in the present model (S1 Fig).

Third, there are many additional bistable and multistable paradigms to consider modeling [6, 33, 34, 112–117]. A first step towards handling these paradigms could be to extend the features included in the Central analysis to spectral-temporal features. These features are probably important for both Shepard tones [112], and speech stimuli [118]. Both stimuli can lead to clear examples of multistability—when the harmonics are evenly split between two possible pitch-change directions [114], and when the same word is repeated, leading to a series of verbal transformations [6, 23, 33, 34]. Note that multistable outputs, as opposed to simple bistable outputs, could be readily computed from our model, because the output of the object-level analysis is a time-frequency mask which, in the present case, we have interpreted heuristically as a bistable response.

Fourth, bistable perception may help us examine differences between conscious and unconscious states [38–41]. The present model may provide a potential baseline upon which to develop computational models of conscious vs. unconscious perception in auditory scenes. Given the indications that correlates of conscious perception arise within an order of several hundred milliseconds [38, 39, 119](but see [120]), and that conscious states appear to be associated with more comprehensive integration of information [39, 119], this present feed-forward model likely reflects early unconscious processing. In a similar vein, there are likely contributions to bistability in areas well past the auditory cortex (e.g. [23, 33, 107]), functions not modeled in the present case. Additional sources of bistability such as top-down, predictive error signals [5, 7] could have more of a role to play in any consciously driven aspects of perceptual bistability [39, 85].

Fifth, one implication of Fig 3 is that whether adaptation or inhibition is necessary to generate bistability depends on the stage of analysis: the Peripheral stage required both adaptation and inhibition, the Central stage did not require adaptation, and the Object did not require inhibition. (Note that a systematic removal of noise was not possible with the current design: even without the introduction of a noise term, the signal processing of the Peripheral and Central stages introduces apparent noise and the Object stage clustering is probabilistic.) The implication of the cross-stage differences in the requirements for adaptation and inhibition is that the representation over which these terms operate is vital: future work could systematically explore how aspects of the signal representation determine whether adaptation and inhibition are necessary to induce a plausible form of perceptual bistability.

These numerous potential elaborations of the model will benefit from our work here, in that it establishes that several foundational components of perceptual bistability can be distributed throughout a larger system capable of segmenting simple auditory scenes. In particular, because this model consumes raw acoustic input, this will allow future elaborations of the model to be validated across multiple experimental paradigms and stimuli.

### Conclusion

The present computational study indicates that auditory bistability can plausibly emerge from a multitude of different sources, from very early following the auditory periphery to later, object-level encodings of the stimulus. Given the pervasiveness of adaptation, inhibition and, arguably [121], noise, throughout the nervous system, this suggests that bistability arises from these sources throughout the auditory hierarchy. The results also indicate that there are more variations of the later stages consistent with human data than earlier stages of processing. This could suggest that, while distributed, there is a more dominant role for Object-level sources of bistability. Furthermore, when interpreted in light of the significant individual differences found in perceptual bistability [24–32, 96], it is possible that there is greater variability in bistable function for the higher-level stages of auditory processing, than lower-level stages of processing.

## Materials and methods

### Ethics statement

All participants who contributed empirical data as a part of this report provided written consent to participate in the study. Procedures were approved by the University of Nevada, Las Vegas Office of Research Integrity, approval number 867626.

### Model design

In each model in the ensemble the three stages of analysis are a Peripheral analysis, *P*(*t*), a Central analysis, *C*(*t*), and an Object analysis, *O*(*t*)—shown in Fig 1A. Into each level we apply adaptation, *a*(*t*), inhibition, *b*(*t*), and noise, *n*(*t*)—collectively denoted *F*[*x*(*t*)], shown in Fig 1A—with varying magnitudes.

All model parameters are described in Tables 1 and 2. Unless otherwise stated, these parameters were manually selected, to provide a reasonable fit to human data while minimizing computational cost. This occurred during a series of pilot model simulations. Minimal adjustments to these parameters were applied during piloting, to avoid model over-fitting, and we carefully explored the effect of changing a number of these parameters during our analysis (see Sensitivity analysis).

**Table 1:** Model parameters. Some of these took on multiple values. Multiple, consecutive integers are denoted with “…”.

	value	description	Eqs
*f*	$440\text{Hz}\times 2^{\frac{-1\ldots 17-0.5}{12}}$	frequency channels	1–4
*ω*	2^−1…2^ cycles/octave	frequency-scale channels	6–8
*ψ*	2^1…5^Hz	temporal-rate channels	10
*r*	2	components of NMF factorization	11
*T*	30	Object-level history	13
*σ*_*F*_	5 log Hz	object continuity neighborhood	14
*κ*	15*s*/Δ*t*, 20*s*/Δ*t*	source prior, mean degrees of freedom	15
*α*	1	source prior, shape for variance	16
*β*	0.25, 0.5, 0.8	source prior, scale for variance	16
*Z*_*α*_, *Z*_*β*_	2, 2	Bernouli prior for object presence	18
*c*_*a*_	values shown in Figs 3–4	magnitude of adaptation	21
*τ*_*a*_	3 s	time constant of adaptation	22
*c*_*b*_	values shown in Figs 3–4	magnitude of inhibition	23
*τ*_*b*_	0.35 s	time constant of inhibition	24
*θ*_*b*_	6	strength of inhibition neighborhood	25
*c*_*n*_	0.2	magnitude of noise	26
*τ*_*n*_	500 ms	time constant of noise	

**Table 2:** Model parameters that varied by level of analysis.

	Peripheral	Central	Object	description	Eq.
Δ*t*	20 ms	20 ms	100 ms	analysis time step	1, 6, 13
Σ_*b*_	5.6	15	$7s\underset{0}{/}\Delta t\;\underset{3}{0}$	inhibition breadth	25
*x*_*α*_	0.005	0.005	0	lower input bound	27
*x*_*ω*_	5	0.1	1.0	upper input bound	27

To assist in the perusal of the model description, Table 3 provides a list of the most important functions, and their meaning. In cases where a variable is left out of a function (e.g. *x*(*t*) instead of *x*(*t*, *f*)) the implication is that the output is multi-valued (e.g. *x*(*t*) = [*x*(*t*, *f*_1_), *x*(*t*, *f*_2_), …]).

**Table 3:** Major functions and their meaning.

	description
*F*[*f*(*t*)] or *X*^*F*^	Application of adaptation, inhibition and noise to *f*(*t*) or *X*
*a*(*t*)	adaptation
*b*(*t*)	inhibition
*n*(*t*)	noise
*x*(*t*)	input function to *F*[*x*(*t*)]
*y*(*t*)	output of *F*[*x*(*t*)]
*P*(*t*, *f*)	Peripheral analysis, of time *t* and frequency *f*
*C*(*t*, *f*, *ω*)	Central analysis, of scale *ω*
*O*(*t*, *f*, *h*)	Object analysis, of source *h*
*K*(*t*, *j*)	component *j* of temporal coherence output
*g*(*t*, *i*, *h*)	source *h* of interpretation *i* of object continuity output

#### Analysis stages

The three analysis stages of each model in the ensemble are a Peripheral (Fig 1A; left panel), Central (middle panel) and Object (right panel) analysis. Each analysis stage outputs a series of time slices separated by Δ*t* (c.f. Table 2).

The Peripheral analysis (Fig 1A; left panel) reflects processing that occurs shortly after and at the periphery of the auditory system (e.g. Cochlear Nucleus) as described in [69]. It is similar in concept to a log-frequency spectrogram, but aims for a more physiologically realistic functional form: first, cochlea-like filter shapes *h*_*f*_ are applied to the original 8000-Hz, time-amplitude signal *x*(*t*) (Eq 1), followed by local inhibition (Eq 2; with $P_{f}^{L}\left(t\right)=0$ for *f* = 0). This is followed by half-wave rectification [a]+, and a low pass filtering of each frequency channel, *h*_*lp*_ (Eq 3). In this case, the low-pass filter is a first order IIR (infinite impulse response) with a -3 dB cutoff frequency of approximately 10 Hz. Finally, adaptation, inhibition and noise (*F*[*x*]) are applied along the frequency channels *f* (Eq 4).

$$\begin{array}{c} P^{H}\left(t,f\right)=x\left(t\right)*h_{f}\left(t\right) \end{array}$$ (1)

$$\begin{array}{c} P^{L}\left(t,f\right)=P^{H}\left(t,f\right)-P^{L}\left(t,f-1\right) \end{array}$$ (2)

$$\begin{array}{c} P(t,f)={\left[P^{L}\left(t,f\right)\right]}^{+}*h_{lp}\left(t\right) \end{array}$$ (3)

$$\begin{array}{c} P^{F}\left(t,f\right)=F_{f}\left[P\left(t,f\right)\right] \end{array}$$ (4)

The Central analysis (Fig 1, middle panel) reflects processing that occurs in the inferior colliculus (IC) [78–81] and primary auditory cortex (A1) [69, 82, 83]. There are quite a number of proposed feature dimensions (e.g. pitch [122], location [123]) processed sometime after the initial peripheral analysis. Here, given the nature of our stimulus and task, we focus on a single dimension: spectral scale. While other features are likely relevant, this single feature can capture a number of useful properties important for our analysis here. Following Chi et al [69], spectral scale is captured via a wavelet transform of the auditory spectrogram along the frequency axis, using the following seed function:

$$\begin{array}{c} H_{\omega }\left(f\right)=\omega \left(1-{\left(\omega f\right)}^{2}\right)e^{-\frac{{\left(\omega f\right)}^{2}}{2}} \end{array}$$ (5)

This is translated into the final complex-valued filter *h*_*ω*_(*f*), using a Hilbert transform, as described in [69]. The result of this scale analysis is a sequence of complex-valued spectrograms (Eq 6), each reflecting the amplitude and phase response to the spectrogram at a different spectral scale *ω* (in Fig 1, middle panel, the amplitude of these responses are shown). The larger the scale the more blurry the resulting spectrogram. Adaptation, inhibition and noise (*F*[*x*]) are then applied by first computing the average magnitude for each scale (Eq 7), and rescaling by these magnitudes (Eq 8).

$$\begin{array}{c} C(t,f,\omega )=P^{F}\left(t,f\right){*}_{f}h_{\omega }\left(f\right) \end{array}$$ (6)

$$\begin{array}{c} C^{S}\left(t,\omega \right)=\frac{1}{N}\sum_{f}C\left(t,\omega \right) \end{array}$$ (7)

$$\begin{array}{c} C^{F}\left(t,f,\omega \right)=C\left(t,f,\omega \right)\frac{F_{\omega }\left[C^{S}\left(t,\omega \right)\right]}{C^{S}\left(t,\omega \right)} \end{array}$$ (8)

In Eq 7, *N* is the number of frequency bins (c.f. Table 1, first row).

The Object analysis reflects computations that occur during the segmentation of sounds into objects (also called streams): it computes multiple, probabilistic groupings of the features in a sound scene and selects one of them (Fig 1B; right panel). It is a novel implementation, similar in spirit to some existing, more abstract models of probabilistic scene interpretation [64, 93]. Its output is a series of time-frequency spectrograms of each sound source. Two basic principles guide the grouping of features into objects: first, we employ temporal coherence [70, 84, 85, 94]—the notion that features which change at the same rate likely originate from the same object—second, we employ object-continuity [9, 71, 86, 87]—the notion that, all else being equal, features originating from the same object tend to change smoothly in time. There are two stages of computation: a near-simultaneous and a sequential grouping stage.

In the first stage of computation, a set of near-simultaneous groupings of features are found by using the principle of temporal coherence. Specifically, a set of components, *K*(*t*), are found using a non-negative factorization of each time window *w*(*t*) across many temporal rates of the features of *C*(*t*), the output of the Central analysis. This factorization of different temporal rates allow us to compute features that consistently change in a synchronous manner across the window of analysis *w*(*t*). First, we apply a series of temporal modulation filters across the central level analysis (Eq 9).

$$\begin{array}{c} R(t,f,\omega ,\psi )=C^{F}\left(t,f,\omega \right){*}_{t}h_{\psi }\left(t\right) \end{array}$$ (9)

These filters are analogous to the spectral filters used to compute *C*(*t*): they each extract a distinct rate of change (amplitude modulation), and are described in [69]. Then, across a limited time window *w*(*t*), we represent absolute values in *R* as a matrix *M*, with the rows as slices of time and temporal modulation, and the columns as slices of frequency and scale (Eq 10). We then find a low rank *r* positive matrix approximation of *M* (Eq 11). We solve Eq 11 using the algorithm from [124].

$$\begin{array}{ccc} M_{ij}\left(w\left(t\right)\right)=|R\left(t_{i},f_{j},\omega_{j},\psi_{i}\right)| & \text{where}\; & t_{i}\in w\left(t\right) \end{array}$$ (10)

$$\begin{array}{ccc} M(w(t))\approx W(t)\times K(t) & \text{where}\; & W(t),K(t)\geq 0 \end{array}$$ (11)

In the second stage of computation, a sequential grouping of the components is found by applying the principle of object continuity. Specifically, each interpretation *i*, consists a set of *H* sources (where *H* may differ, frame to frame), with each source denoted as *g*(*t*, *i*, *h*). Each source is defined by a sum of a set of component indices *k*(*t*, *i*, *h*), which index the rows of *K*, denoted by *K*(*t*, *j*) (Eq 12). In our simulations we consider up to *r* sources (c.f. Table 1).

$$\begin{array}{c} g(t,i,h)=\sum_{j\in k(t,i,h)}K\left(t,j\right) \end{array}$$ (12)

We solve for the set of components *k*(*t*, *i*, *h*) belonging to source *h* according to Eqs 12–17 using a simple greedy approach: we compute the solution for each time step *t* sequentially, holding all prior time steps constant. The grouping of components for all sources *g*(*t*, *i*) constitutes a single interpretation, *i*. We found one such interpretation for each pair of values for the hyper-parameters *κ* and *β* (c.f. Table 1); these hyper-parameters determined the degree of feature smoothness assumed by a given interpretation and their role is described below, more precisely.

We model the observation of a source at time *t* as a multivariate Normal distribution consistent with the last *T* observations of that source (Eq 13).

$$\begin{array}{cc} g(t,i,h)|\kappa ,\beta ,g(t-\Delta t,i,h),g(t-2\Delta t,i,h),\ldots g(t-T\Delta t) & ∼Z_{h}\left(t\right)\cdot N\left(\mu_{t},\sigma_{t}^{2}\Sigma \right) \end{array}$$ (13)

Δ*t* is the time step (c.f. Table 1). The integer *Z* (0 or 1) reflects the fact that not all sources would be present at all moments in time. The correlation matrix Σ is key to encoding the principle of object continuity. It is a fixed matrix that had greater correlation when the frequency *f* of component *i* was closer to the frequency of component *j* (Eq 14).

$$\begin{array}{c} \Sigma_{ij}=\exp(-\frac{{\left(\log f\left(i\right)-\log f\left(j\right)\right)}^{2}}{\sigma_{F}^{2}}) \end{array}$$ (14)

This correlation structure therefore encodes the principle that objects will smoothly transition along their frequency components (*σ*_*F*_ is defined in Table 1).

The distribution of *μ* and *σ* determine the strength of the assumption of object continuity, and are conjugate priors for ease of computation (a Normal distribution in Eq 15, and Gamma distribution in Eq 16). The shape of their distributions depends on two variable hyper-parameters: the scale of prior mean, *κ*, and the scale of the variance, *β* (*α* is found in Table 1).

$$\begin{array}{c} \mu_{t}|\kappa ,\beta ∼N_{\tau }\left(0,\sigma_{t}^{2}/\kappa \right) \end{array}$$ (15)

$$\begin{array}{c} \sigma_{t}^{2}|\kappa ,\beta ∼\Gamma (\alpha ,\beta ) \end{array}$$ (16)

The effect of *κ* and *β* are to control the strength of the prior “evidence” relative to the strength of the observed data (the likelihood), because they control the overall span of values that are deemed plausible by the model priors. With a stronger prior (containing a narrower span of plausible values) the model of each source is slower to change in the face of data, and so the resulting posterior favors more slowly moving sources. With a weaker prior (containing a broader span of plausible values) the model of each source changes more quickly, and so the resulting posterior favors more quickly moving sources. In the present case, a prior favoring more slowly moving sources would typically favor a segregated interpretation of the ABA stimulus, because A as a source and B as a source are both completely stationary, while a prior favoring more quickly moving sources would typically favor a fused percept, because the A+B source shows movement between the two frequencies of A and B.

As noted above, the variable *Z* (Eq 17) determines the presence (1) or absence (0) of each object within a given frame. It has a Bernouli distribution, and is given a conjugate prior (a Beta distribution) for ease of computation, with fixed parameters *Z*_*α*_ and *Z*_*β*_ (Eq 18)

$$\begin{array}{c} Z_{h}\left(t\right)∼\text{Bern}(\theta ) \end{array}$$ (17)

$$\begin{array}{c} \theta ∼B(Z_{\alpha },Z_{\beta }) \end{array}$$ (18)

Finally, the dominant interpretation at each time frame (Eq 19) is used to compute the time-frequency mask of each source *O*(*t*, *f*, *h*) (Eq 20). Adaptation, inhibition and noise (*F*[*x*]) are applied to the relative log-probabilities of each interpretation.

$$\begin{array}{c} I(t)=\underset{i}{arg max}F_{i}[c\cdot \text{logpdf}(g(t,i))] \end{array}$$ (19)

$$\begin{array}{c} O(t,f,h)=C^{-1}[C(t)\cdot g(t,I(t),h)] \end{array}$$ (20)

The constant *c* is selected to normalize the log probability distribution function (logpdf) such that the largest value after the first second of output was 1 (the initial logpdf values can be quite erratic, so we avoid values in this first segment). The function *C*^−1^ denotes the inverse of *C*(*t*) and its output is a time-frequency representation in the same format as the Peripheral analysis *P*(*t*).

We interpret the output *O*(*t*, *f*, *h*) as a behavioral response using a heuristic which we describe under Model evaluation.

#### Adaptation, inhibition and noise

Adaptation, inhibition and noise follow a similar functional form as found in [48, 65], and are applied to a set of input weights *x*(*t*) within each stage of analysis, yielding a new set of weights *y*(*t*) (Fig 1B). This overall transformation of weights is denoted using *F*[*x*(*t*)]. The components are applied separately to each unit of an analysis stage: frequencies (for Peripheral), scales (for Central), or object interpretations (for Object). The units are denoted by a subscript *i* in the following equations.

Each term modulates the unsmoothed weights *y*^*U*^(*t*) and is characterized by a magnitude *c*—which determines how much the term modulates the output—and a time constant *τ*—which determines how quickly the term changes.

The amount of adaptation for each weight $y_{i}^{U}\left(t\right)$ is determined by a low-pass, delayed version of $y_{i}^{U}\left(t\right)$ (denoted *a*^*D*^), shown in Eqs 21 and 22

$$\begin{array}{c} a_{i}\left(t\right)=c_{a}a_{i}^{D}\left(t\right) \end{array}$$ (21)

$$\begin{array}{c} {\dot{a}}_{i}^{D}\left(t\right)=\frac{y_{i}^{U}\left(t\right)-a_{i}^{D}\left(t\right)}{\tau_{a}} \end{array}$$ (22)

The dot ($\dot{a}$) above a function is used to denote the first derivative of that function. All functions are solved using a first order, finite-differences approach, using a delta specific to the particular stage of analysis (see Table 2).

The amount of inhibition for each weight $y_{i}^{U}\left(t\right)$ is determined by a low-pass, delayed version of distant neighboring weights, shown in Eqs 23 and 24. The neighbors are selected by column vector *B*_*i*_ of weight matrix *B*, which varied depending on the level of an analysis.

$$\begin{array}{c} b_{i}\left(t\right)=c_{b}b_{i}^{D}\left(t\right) \end{array}$$ (23)

$$\begin{array}{c} {\dot{b}}_{i}^{D}\left(t\right)=\frac{B_{i}\cdot y^{U}\left(t\right)-b_{i}^{D}\left(t\right)}{\tau_{b}} \end{array}$$ (24)

$$\begin{array}{c} B_{ij}=\theta_{b}\{1-\exp[{\left(v\left(i\right)-v\left(j\right)\right)}^{\prime }\Sigma_{b}^{-1}\left(v\left(i\right)-v\left(j\right)\right)]\} \end{array}$$ (25)

Each value in the inhibition weight matrix, *B*_*ij*_, is proportional to the distance between the labels of units *i* and *j*: for the Peripheral analysis this is a scalar value, in log frequency, for the Central it is a scalar in log cycles per octave, and for the Object analysis it is a two dimensional vector, the first term indicating the number of samples of prior data (for *κ*, c.f. Eq 15) and the second term a scale value (for *β*, c.f. Eq 16). These distances are scaled by the distance weighting matrix Σ_*b*_ (c.f. Table 2).

The amount of noise for each weight $y_{i}^{U}\left(t\right)$ is defined by *n*_*i*_(*t*) (Eq 26)

$$\begin{array}{c} {\dot{n}}_{i}\left(t\right)=c_{n}W\left(t\right)-\frac{n_{i}\left(t\right)}{\tau_{n}} \end{array}$$ (26)

The *W*(*t*) term is a Wiener process (a.k.a. Brownian motion).

The three terms are used to modulate a bounded, smoothed version of the input weights (Eq 27) resulting in the initial, unsmoothed output of each weight $y_{i}^{U}\left(t\right)$ (Eq 28).

$$\begin{array}{c} {\dot{\tilde{x}}}_{i}\left(t\right)=\frac{{\tilde{x}}_{i}\left(t\right)-{\left(x_{i}\left(t\right)\right)}_{x_{\alpha }}^{x_{\omega }}}{\tau_{x}} \end{array}$$ (27)

$$\begin{array}{c} y_{i}^{U}\left(t\right)={\left[{\tilde{x}}_{i}\left(t\right)\times \left(1-a_{i}\left(t\right)\right)e^{n_{i}\left(t\right)}-b_{i}\left(t\right)\right]}^{+} \end{array}$$ (28)

In the above equations, [a]+ denotes half-wave rectification (all values below zero are set to 0), and (a)xαxω indicates a soft sigmoid bound between *x*_*ω*_ and *x*_*α*_.

The final output weights are then computed according to Eq 29.

$$\begin{array}{c} F_{i}\left[x_{i}\left(t\right)\right]=y_{i}\left(t\right)=y_{i}^{U}\left(t\right){*}_{t}h_{s} \end{array}$$ (29)

The *_t_ denotes convolution in time with *h*_*s*_, a low-pass Butterworth filter of order 3 at 1.5Hz. The low-pass filter ensured that brief changes in amplitude (due to the silence between tones) did not dramatically change the overall effect of adaptation and inhibition.

### Model evaluation

We evaluate model responses on an ABA stimulus, created to exactly match the stimulus used in Snyder et al [16]. It consists of two different frequencies: one at 500 Hz (A) and another either 3, 6 or 12 semitones (st) above 500 Hz (B). Each triplet consisted of three equally spaced 50 ms tones, with onsets separated by 120 ms. The triplets are separated by 480 ms each. Pure tones included a 10 ms cosine ramp at the onset and offset.

The key model parameters we manipulate during our experiments are the magnitude of adaptation (*c*_*a*_) and inhibition (*c*_*b*_) across the values 0, 5, 15, 44, 130, 130, 390, 1100, 3400, 10000 and 10^5^, for the within-level variations, and across 0, 5, 63, 790 and 10^5^, for the across-level variations. This results in 300 total pairings of the within-stage parameters (10^2^ × 3), and 390, 625 pairings of the across stage models ($5^{2^{3}}$). For each pairing of the within-stage model parameters we run 20 simulations, each with a 48 second stimulus composed of 100 repetitions of the ABA pattern. This is repeated three times, for the three stimulus conditions (3, 6 or 12 st). For each of the across-stage model variations, we use the same procedure, but it terminated early for all poorly performing model variations (Fig 4): following the first 10 simulations of the 6 semitone stimulus (always the first stimulus evaluated), if the model:human deviation ratio is greater than one, no more simulations for that model are run. This procedure ensures that our finite computational resources were not spent evaluating the merits of models that clearly performed poorly on this most essential stimulus condition.

#### Interpretation of model output

To interpret the model output for each simulation, we use the following strategy. During the streaming task human listeners are often asked to respond continuously, reporting if they heard a “fused” (1 stream) or “segregated” percept (2 or more streams). To obtain a similar response for the model we interpret the maximum amplitude time-frequency mask found at each frame of *O*(*t*) as a “fused” or “segregated” response using an automated heuristic. Specifically, we compare the estimated frequency bandwidth of this dominant mask to that of the input’s time-frequency representation, *P*(*t*), and reported a 2 stream percept only if this ratio was greater than Θ_*r*_ (c.f. Table 4).

**Table 4:** Heuristic parameters (c.f. Eq 30).

	value	description
Θ_*r*_	0.75	heuristic threshold ratio
Θ_*c*_	0.996	heuristic global threshold
Θ_*f*_	0.95	heuristic local threshold
Θ_*w*_	500 ms	heuristic time window
Θ_Δ_	250 ms	heuristic time step

To estimate the frequency bandwidth, we use the following procedure. The frequency bandwidth of the dominant source is found for multiple overlapping windows of analysis *w*_*b*_(*t*). For each time window (of length Θ_*w*_, at time step Θ_Δ_) this bandwidth is estimated by finding the maximum distance between frequency channels whose $\Theta_{f}^{\text{th}}$ quantile falls above a data-driven threshold, determined by the $\Theta_{c}^{\text{th}}$ quantile of the entire mask. Specifically, using *Q*_*f*_(*p*) to denote the *p*^*th*^ quantile of frequency channel *f*, we estimate the bandwidth as follows

$$\begin{array}{c} \max_{f,g}\left|f-g\right|\;\text{where}\;f,g\in \{x|Q_{x}\left(\Theta_{a}\right)>\Theta_{b}\cdot Q_{P(t)}\left(\Theta_{c}\right)\} \end{array}$$ (30)

The values for all Θ parameters (shown in Table 4) are selected by hand to provide model responses consistent with a visual inspection of sampled output masks. We eliminate any brief switches: any response that returns back to the original response within 250 ms is discarded as a spurious output of our decision-making heuristic.

Before analyzing the model responses during the computation of our model:human deviation ratio, we discard the first response. This same procedure has been used on human data to examine the steady state behavior, to avoid any “buildup” in the first response to the stimulus [4], a feature our model appears to replicate (S1 Fig).

### Human data

We use human data from three sources: [16, 88] and a new group of participants. From the two existing data sources, we use the context phase of Experiment 1A of Snyder et al [16] and the context phase of Experiment 1 and 2 of Yerkes et al [88]. The stimuli are identical to those described above, but the base frequency was 300 Hz instead of 500 Hz in some of the conditions (the measures of interest here did not appear to differ across these two different frequencies). We found the proportion of segregated responses by selecting the current response during the time range of 4 to 6 seconds for each individual. If multiple responses occurred during this window, a weighted average was calculated, based on the proportion of time spent on a given response within the 4 to 6 second window. This window was selected to fall within a steady state period of the experimental stimulus on the basis of Fig 1 of [16]. Before this steady state period listeners tend to be biased towards fused percepts, and gradually transition to a given balance between fused and segregated responses in a manner specific to the stimulus employed, a process called “buildup”. Note that the rate of buildup in [16] differs from several other recent studies [65, 91, 92]), probably due to variations in stimulus and/or experimental design. When estimating the distribution of individual percept lengths across all listeners (Fig 2B), we followed the same procedure used for the model output: we removed the first percept and treated any responses less than 250 ms long as spurious (this latter step eliminated %1.2 of the data).

The new group of listeners included a total of 35 normal-hearing participants (22 females) with an average age of 24.5. Listeners were recruited from the University of Nevada, Las Vegas community and paid for their participation. During the condition documented here, participants were presented the stimulus described above with a base frequency of 400 Hz, presented over 3M E-A-RTONE insert ear phones in a sound attenuated booth, at a level of 60 dB. There were a total of 24 trials broken into three 8-trial blocks. Each trial of the stimulus was 48 seconds long, consisting of a total of 100 repetitions of the ABA sequence. For the duration of each trial, listeners were asked to continuously report whether they heard fused or segregated percepts. Participants began the experiment with a short practice session during which the galloping rhythm of the fused percept, and the more steady rhythm of the segregated percept were explained.

### Statistical analyses

All reported confidence intervals are computed using a non-parametric bootstrap [125] with 10,000 samples. The density curves shown in Fig 2B were computed using a method for log-space density estimation [126] with a Normal kernel, where kernel bandwidths were determined by the Silverman rule [127].

## Supporting information

S1 Fig Early behavioral responses of four example models. The four models are the same as those shown in Fig 2C. The percent-streaming (y-axis) is computed for a total of N = 1000 simulation runs of each model (columns) across the three stimuli (colors and line styles) over the first 10 seconds (x-axis).
(TIF)
