# Supplementary figures and images for: Ensemble modeling of auditory streaming reveals potential sources of bistability across the perceptual hierarchy
Source: PLoS Comput Biol. 2020 Apr 10;16(4):e1007746. doi: 10.1371/journal.pcbi.1007746 (PMC7185718; doi:10.1371/journal.pcbi.1007746)

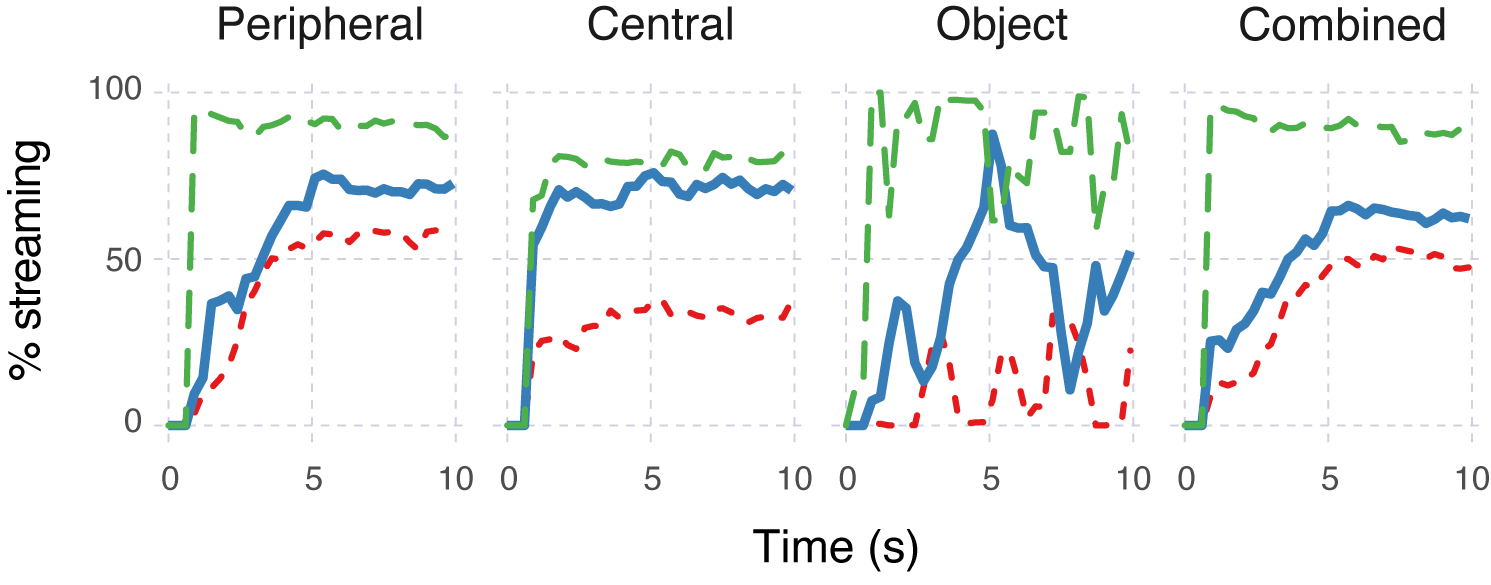

Supplement: S1 Fig — The four models are the same as those shown in Fig 2C. The percent-streaming (y-axis) is computed for a total of N = 1000 simulation runs of each model (columns) across the three stimuli (colors and line styles) over the first 10 seconds (x-axis). (TIF) [file pcbi.1007746.s001.tif]
